# Anodal tDCS over the right parietal but not frontal cortex enhances the ability to overcome task set inhibition during task switching

**DOI:** 10.1371/journal.pone.0228541

**Published:** 2020-02-18

**Authors:** Stefano Sdoia, Pierpaolo Zivi, Fabio Ferlazzo

**Affiliations:** Department of Psychology, Sapienza University, Rome, Italy; University of Groningen, NETHERLANDS

## Abstract

Switching between tasks requires individuals to inhibit mental representations of the previous task demands and to activate representations of the new task demands. The inhibition of the executed task remains active for a while so that when the inhibited task set must be re-activated shortly after, the need to overcome residual task set inhibition leads to behavioral costs. In a sham-controlled balanced-order within-subjects experimental design we investigated whether applying right anodal/left cathodal transcranial direct current stimulation (tDCS) over the dorsolateral prefrontal or parietal cortex modulated the ability to overcome persistent task inhibition during task switching. Results showed that right anodal/left cathodal tDCS over the parietal cortex improves performance selectively when switching back to a recently inhibited task that requires previous inhibition to be overcome. Right Anodal/left cathodal tDCS over the prefrontal cortex improves performance during task switching in general, either when re-engaging in a inhibited task or when engaging in a non-inhibited task. Results suggest a different contribution of prefrontal and parietal regions to task switching, with parietal cortex being selectively involved in overcoming persistent task inhibition and prefrontal cortex being more generally involved in the control of task set during task switching.

## Introduction

The ability to flexibly adjust behavior to a changing environment by promoting the processing of current goal-relevant information at the expense of the no longer relevant one is a key factor for efficient adaptation and survival, particularly when irrelevant information interferes with current intention, eliciting conflicting responses. Behavioral adaptation to external changes is supported by cognitive control processes, a set of neurocognitive mechanisms that, based on current goals establish internal constraints on the way we process external information, defining—for instance—the information to attend to at the perceptual level (e.g., the color), at the motor level (left hand movement), and the association between potential stimuli and responses (e.g., if red press the left button). Thus, cognitive control grants behavioral flexibility by establishing and strategically modifying the task set, that is the transient and arbitrary associations between mental representations of stimuli and responses in accordance with current goals (i.e., task set; [1]).

Response adaptation to changing task demands has been often studied in laboratory by means of the task switching procedure, wherein participants typically alternate between performing each of two or more possible tasks afforded by the same stimulus (see [2,3] for reviews). In this procedure, the control settings appropriate for one task become no longer relevant when a new task is required, so that cognitive control is necessary for the instantiation of the appropriate task set (e.g., defining the new relevant information at perceptual and motor levels). The need to reconfigure the internal control settings required to perform a new task is considered a source of the so-called *switch cost* [4–6], that is the reaction time (RT) difference that typically results from the slower performance on trials where the participant has to switch to a different task (switch trials) compared to trials where the participant has to repeat a task (no-switch trials). Interestingly, no-longer-appropriate task sets remain active after their instantiation [7–11], interfering proactively with the new task set, so that when rapidly shifting from one task to another inhibition could be necessary to counteract this persistent activation and to switch efficiently to the new task (see [12] for a review). Importantly, the inhibition of the executed task remains active for a while so that when the inhibited task set (e.g., task A) is reactivated shortly after, as in an A–B–A task sequence, it is unlikely that it has fully recovered from previous inhibition. The need to overcome this suppressed state leads to a behavioral cost, named n-2 task repetition cost, which has been demonstrated by showing that switching back to a task that has been executed very recently (e.g., A-B-A task sequences) is harder than switching back to task that has been executed a less recently (e.g., C-B-A task sequence; e.g., [13,14]).

This form of inhibition (also known as backward inhibition; [13]) has attracted interest in cognitive psychology mostly because it seems to target high-level mental representations, such as the whole task set, rather than individual perceptual features (e.g., red color; e.g., [15]) or motor responses (e.g., left-hand button press; e.g., [16]). On these grounds, task inhibition has been subject to intense research in cognitive psychology, but its neural mechanisms remain unclear.

Neuroimaging studies consistently suggest that both frontal and parietal regions play a crucial role in task switching (e.g., [17–23]) but the individual contribution of these brain regions to task set inhibition has remained largely uninvestigated. Dreher & Berman (2002) [24] reported larger activity in the right lateral prefrontal cortex, as assessed by functional magnetic resonance imaging, when switching to a task recently performed compared when switching to a task less recently performed (i.e., ABA versus CBA task sequence), and suggested that the right prefrontal cortex plays a role in overcoming task inhibition. Consistent with this result, a reduced task inhibition was also reported in participants with damage to the right, but not to the left, lateral prefrontal cortex [25]. However, larger activity during task switching was also reported in other brain regions in participants who were good at inhibiting previous task sets, such as the basal ganglia and supplementary motor area/premotor area, compared to participants who were less good at inhibiting an irrelevant task [26]. On the other hand, electrophysiological studies consistently reported increased negativity at parietal sites when switching back to a recently executed task [27–28] suggesting that the parietal cortex also plays a role in task inhibition. Hence, findings from neuroimaging and electrophysiological studies reported modulation of brain activity at both frontal and parietal sites related to inhibition of irrelevant task set, and converging evidence are still needed to clarify the specific contribution of these regions.

Insight into the neural mechanisms of task set inhibition can be obtained by actively manipulating the neural activity of specific brain regions that are supposed to be involved in task inhibition and assessing the impact of this perturbation on behavioral performance (i.e., n-2 task repetition cost). One possibility to non-invasively modulate the cortical excitability is offered by transcranial direct current stimulation (tDCS) [29]. tDCS allows transient modulation of spontaneous neuronal excitability through the delivery of a low constant electric current flow through two electrodes applied to the scalp. This electric current flow alters the polarization of the resting membrane potential, such that cortical excitability is increased in the region below the anode electrode, and decreased in the region below the cathode electrode [30–32]. The goal of the present study was to investigate whether applying tDCS at frontal and parietal sites modulates the ability to overcome the persistent inhibition during task switching, as assessed by the n-2 task repetition cost. Specifically, in a sham-controlled, balanced-order within-subject experimental design, right anodal/left cathodal tDCS was applied over prefrontal or parietal scalp sites during a task switching procedure. The mean accuracy and reaction times (RTs) were recorded to assess performance on trials wherein participants had to switch back to a previously inhibited task (ABA switch sequence), wherein they had to switch back to a non-inhibited task (CBA switch sequence), and wherein they had to repeat the same task they performed on the previous trial (AA no-switch sequence).

## Materials and methods

### Participants

20 healthy subjects with a mean age of 26.3 years (s.d. 3.64; 12 women) participated in the study. All participants reported normal or corrected-to-normal vision, no history of neurological or psychiatric disorders, and no ongoing medication. They all were naïve to the aims of the study. The sample size was defined through power analysis, using a medium to large partial eta^2^ of 0.2 for the higher order interaction and a power of 0.90 to increase the chance of replicability. The study was approved by the Ethics Committee of the Department of Psychology at the Sapienza University and conducted in accordance with its policies. All participants provided written informed consent.

### Procedure

Task cues were black geometrical frames (a square, a diamond, and a circle) with a size of about 6 cm by 6 cm, centrally presented on a grey background. Stimuli were digits from 1 to 9, except for the digit 5 that was never presented. Each digit was about 2 cm in height and 1cm in width, and was centrally presented, superimposed on the task cue.

Participants performed three different numerical judgment tasks: a magnitude task, requiring participants to indicate whether the digit was smaller or larger than five; a parity task, requiring participants to indicate whether the digit was an odd or an even number; and a position task, requiring participants to indicate whether the digit was centrally or peripherally positioned along the number line (3, 4, 6, and 7 were considered central digits; 1, 2, 8, and 9 were considered peripheral ones). The magnitude task was cued by the diamond, the parity task by the square, and the position task by the circle.

Participants responded by pressing the A key of a standard QWERTY keyboard to the smaller-than-5, even, and centrally positioned digits, and with the L key to the larger-than-5, odd, and peripherally positioned digits.

Participants were tested individually in a dimly lit testing room. The cues and the stimuli were centrally presented on a 17-inches computer monitor (refresh rate: 60 Hz) placed 60 cm from the participant. The experiment was programmed in E-Prime on a computer running the Microsoft Windows XP operating system.

Instructions about the tasks, the cue-task associations, and the category-response associations were displayed on the screen and verbally detailed to each participant at the beginning of the experiment.

The experiment consisted of four blocks of 96 trials each. On each trial, the task cue was presented first. After 600 ms, the stimulus was presented over the task cue. Participants were required to respond to the stimulus as fast as possible, according to the task rules indicated by the task cue. In case of an error, an auditory error feedback was provided for additional 50 ms. Error feedback was also provided for reaction times slower than 2500 ms. The experiment started with the participant pressing the space bar.

Task sequences were pseudo-randomized with the constraints of having approximately 100 ABA switch trials, 100 CBA switch trials, and 100 AA no-switch trials. No-switch trials were included as a control condition, as we expected the tDCS to selectively affect performance on switch trials (ABA and CBA) and not on no-switch trials, and also to reduce potential expectancy-related effects due to having only switch trials.

### Transcranial direct current stimulation

In three separated task-switching sessions one week apart, all participants underwent three different right anodal/left cathodal stimulation conditions: frontal, parietal, and sham tDCS during the task performance (online stimulation). In the frontal stimulation condition the anode was placed over the right dorsolateral prefrontal cortex (F4 according to 10–20 EEG International System) whereas the cathode electrode was placed over the left dorsolateral prefrontal cortex (F3). In the parietal stimulation condition the anode was placed over the right parietal site corresponding to P4 and the cathode electrode over P3. In the sham condition, electrodes placement was the same of the frontal condition. Session order was randomized across participants. During the two active sessions, a direct current of 1.5 mA was induced by two saline-soaked circular sponge electrodes (3 cm diameter, density 0.2 mA/cm2) and delivered by a battery-driven constant current stimulator (BrainStim E.M.S., srl Bologna, Italy) with a fade in/fade out ramp of 45 s. In the sham condition, the stimulation only involved the fade in/fade out phase and 2 seconds of stimulation.

## Results

Mean individual reaction times (RTs) and error rates (ERs) were analyzed in a 3 X 2 repeated measures ANOVA design using Stimulation (frontal, parietal, and sham) and Sequence (ABA and CBA) as independent variables. The first block was considered as practice and excluded from the analyses. Only ABA and CBA task sequences with correct responses on trials *n*, *n–* 1, and *n–* 2 were included in the RTs analyses. One participant was excluded from the analyses because of poor task accuracy and extremely slow RTs (percent of correct responses was more than two standard deviations below the group mean and the RTs average was more than two standard deviations above the group mean in the frontal session). Mean RTs and ERs for all the conditions are reported in Table 1.

**Table 1:** Mean reaction times, accuracy and n-2 task repetition costs as a function of the stimulation (sham, frontal and parietal) and the task sequence (ABA and CBA). Standard errors are in brackets.

Stimulation condition	RTs				n-2 task repetition cost (RTs)	Accuracy				n-2 task repetition cost (Accuracy)
	Task Sequence					Task Sequence				
	ABA		CBA			ABA		CBA		
Sham	875	*(31)*	824	*(40)*	50	0,90	*(0*,*01)*	0,91	*(0*,*01)*	-0,01
Frontal	830	*(40)*	769	*(41)*	61	0,92	*(0*,*01)*	0,93	*(0*,*01)*	-0,01
Parietal	830	*(43)*	837	*(51)*	-7	0,90	*(0*,*02)*	0,91	*(0*,*02)*	-0,01

For RTs data, the ANOVA revealed a significant main effect of the Sequence (F(1, 18) = 12.987, p = 0.002, η_p_^2^ = 0.419), showing slower RTs for sequences ABA (846 ms) compared to CBA (810 ms), indicating that a significant *n* − 2 repetition costs occurred. The main effect of the Stimulation was not significant (F(2, 36) = 0.576, p = 0.567). Importantly, the Sequence by Stimulation interaction turned out to be significant (F(2, 36) = 4.137, p = 0.024, η_p_^2^ = 0.187), indicating that the n-2 repetition cost was modulated by the tDCS. Specifically, Duncan post-hoc tests revealed that the RTs were significantly shorter during the right anodal/left cathodal tDCS of the prefrontal cortex than during the sham stimulation on both the ABA (p = 0.025) and CBA sequences (p = 0.004). This indicated that tDCS over the frontal cortex affected the performance on trials where participants switched back to an inhibited task set as well as where they switched to a non-inhibited tasks. Furthermore, Duncan post-hoc test also showed that the RTs on the CBA sequences during the right anodal/left cathodal tDCS of the parietal cortex were not significantly different from those observed during the sham stimulation (p = 0.535). Interestingly, the RTs on the ABA sequences during the right anodal/left cathodal tDCS of the parietal cortex were significantly shorter than those of the ABA sequence during the sham stimulation (p = 0.027). This indicated that tDCS over parietal cortex affected selectively the performance on trials where participants switched back to an inhibited task.

**Fig 1:**
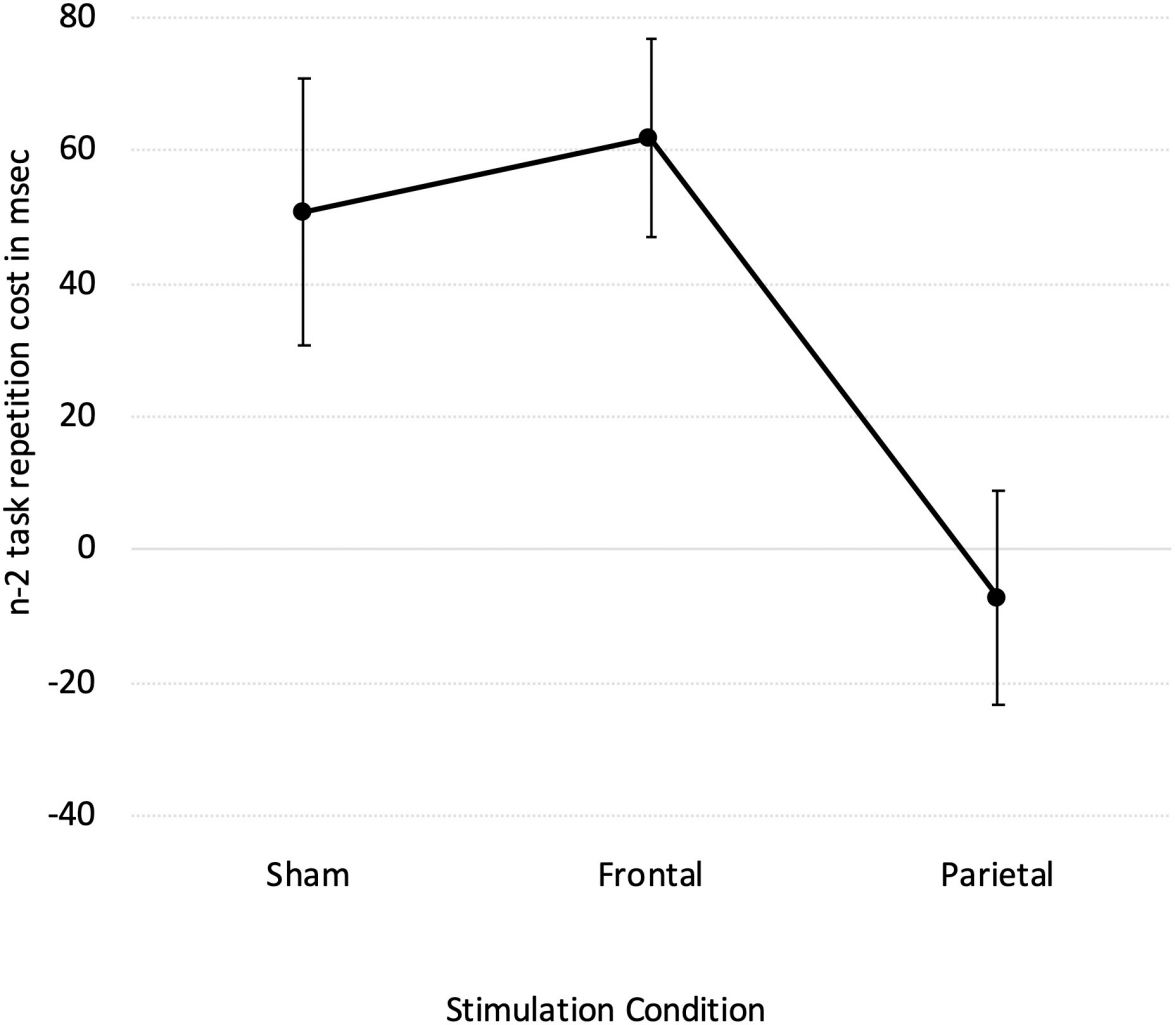
n-2 task repetition costs as a function of the tDCS conditions (Sham, Frontal, Parietal). Bars denote standard errors.

To further specify tDCS modulation of performance we also tested for change in the size of the n-2 task repetition cost in a one-way ANOVA according to the stimulation condition (Sham, Frontal and Parietal; see Fig 1). The significant effect of the stimulation condition (F(2, 36) = 4.137, p = 0.024) revealed that there were no significant differences between the n-2 task repetition cost under the sham and frontal tDCS (51 and 62 msec, respectively; p = 0.668 Duncan test). Importantly, the n-2 task repetition cost during parietal tDCS (- 7 msec) was significantly different from the n-2 task repetition cost during both sham (51 msec) and frontal (62 msec) tDCS (parietal vs sham: p = 0.031; parietal vs frontal: p = 0.015). This indicated that the right anodal/left cathodal tDCS of the parietal cortex significantly reduced the n-2 task repetition cost.

We also analyzed the effects of tDCS on the repetition trials in a one-way ANOVA in order to ruled out the hypothesis that tDCS affected the performance in an unspecific way, for instance by globally reducing or increasing the RTs regardless of the switch or repetition requirements. Results revealed that RTs were the same on sham, frontal and parietal tDCS (F(2, 36) = 0.695, p = 0.505), indicating that tDCS had no effects on no-switch trials.

The ANOVA on mean individual error rates did not reveal any significant main effect or interaction (Stimulation: F(2, 36) = 1.372, p = 0.266; Sequence: F(1, 18) = 1.323, p = 0.265; Stimulation X Sequence: F(2, 36) = 0.101, p = 0.903), indicating that the accuracy was the same regardless of the type of stimulation and the type of task sequence.

## Discussion

In the present study we investigated whether applying right anodal/left cathodal tDCS over the dorsolateral prefrontal or parietal cortices improves the ability of overcoming persistent task inhibition during task switching.

Results revealed that right anodal/left cathodal tDCS of the dorsolateral prefrontal cortex did not affect the ability of overcoming persistent task inhibition. Indeed, tDCS of the prefrontal cortex determined a general improvement when a task switching was required compared to when the same task was repeated, but the size of this improvement was the same either when re-engaging an inhibited task, and thus the previous inhibition had to be overcome, or when switching to a task that did not suffer from previous inhibition. Importantly, right anodal/left cathodal tDCS of the parietal cortex improved the performance selectively when re-engaging a previously inhibited task, without affecting the performance when switching to a task that did not suffer from previous inhibition, eliminating the n-2 task repetition cost completely (Fig 1). This suggests that the parietal cortex has a role in overcoming persistent inhibition of a previously executed task when re-engaging it. Of importance, neither the frontal nor the parietal tDCS affected the performance on the no-switch trials, indicating that the effects of the tDCS over the prefrontal and parietal cortices did not determine a general performance modulation, but selectively influenced the processes involved in task set switching.

The findings that prefrontal stimulation improved the task switching ability and that this improvement equally affected the performance when switching to a non-inhibited task as well as when re-engaging an inhibited task (without affecting no-switch trials) support the idea that the prefrontal cortex has a role in cognitive control processes involved in task set switching, and they are consistent with previous imaging and ERPs studies. The role of prefrontal cortex in task switching has been largely documented (see [33] for a review), and neuroimaging studies have shown prefrontal activations across a variety of stimuli and paradigms (see [34] for a meta-analysis). The lateral PFC activity has been frequently interpreted as reflecting transient cognitive control operations associated with task switching, such as the endogenous task-set reconfiguration [21]. Indeed, the prefrontal cortex has been suggested to exert top-down control to maintain or to update task representations [23,35,36]. Importantly, the present findings suggest that the role of prefrontal cortex is not selectively related to the ability of overcoming persistent task inhibition. Although caution is necessary when comparing findings from different techniques, this result appears inconsistent with the fMRI result reported by Dreher & Berman (2002) [24], who found that the right lateral prefrontal cortex was more activated when switching back to a task recently performed compared to a task less recently performed. However, methodological differences exist between the experimental procedure used by Dreher & Berman (2002) [24] and our present procedure that may explain the different results. For instance, the experimental procedure used by Dreher and Berman (2002) [24] involved the presentation of triplets of tasks that were constructed with the constraints of having only three possible task sequences, namely the ABA, CBA and BAA, instead of a randomized sequence of tasks. Presenting stimuli in specific triplets allowed to maximize the occurrence of task sequences that are relevant for the intended comparisons (e.g., ABA and CBA) but it could incidentally induce implicit expectancy about the identity of the upcoming task or about the sequence of task presentation. For instance, due to the heuristic of representativeness [37], in a situation where three tasks are possible and the tasks are presented in separated triplets of trials, people may judge on each triplet the CBA sequence as more probable than the ABA sequence of tasks. This may induce participants to expect the CBA triplets more than the ABA triplets or to expect a n-2 task switching (i.e., CBA) more than a n-2 task repetition (i.e., ABA). If that were the case, the ABA task sequence would also represent a violation of an implicit expectancy and thus, the prefrontal activation reported by Dreher and Berman (2002) [24] could reflect an expectancy-related effect. Consistent with this hypothesis, evidence has been recently provided that internally generated predictions about the likelihood of a change in task demand are represented in dorsolateral prefrontal cortex [38]. In the present study, this type of expectancy-related effects can be ruled out because, unlike the procedure used by Dreher & Berman (2002) [24], we used a cued-task switching procedure where the occurrence of a task cue on each single trial informed participants about the identity of the upcoming task before each stimulus presentation and without uncertainty; most importantly, each trial was presented one after another and in a randomized order of task presentation, so that the effects that incidentally may induce the occurrence of specific sequences of tasks can be controlled for.

Crucially to the goal of the present study, right anodal/left cathodal tDCS of the parietal cortex improved the performance only when re-engaging in a task that has been recently inhibited, without affecting the performance when switching to a task that did not suffer (or suffered less) of previous inhibition. This suggests that the parietal cortex has a specific role in overcoming task set inhibition during task switching. Prior findings provide support to this idea. The parietal cortex has been found to be consistently activated during task switching (compared to task repetition) in fMRI studies [17–21,23,39,40]. A common region of the superior parietal lobule has been also identified as a source of cognitive control during shifts between perceptual, mnemonic, and rule representations, indicating that the parietal lobe plays a domain-independent role in the instantiation of a new task set [41]. This domain-independent feature is what would be required to a brain structure that is supposed to be involved in inhibitory control and that operates at the level of the whole task set representation rather than on individual stimulus or response features. The superior parietal cortex was also found to be more active for bivalent than for univalent stimuli [18], that is when stimuli elicit multiple competing tasks, and the need for inhibition is strong, than when stimuli are uniquely associated with a single task and there is no task interference and, thus, no need of task inhibition. The domain-independent feature, together with the high neural activity for bivalent stimuli strongly suggests the parietal cortex as a potential candidate for hosting neural population involved in overcoming persistent task inhibition.

Converging evidence to the involvement of parietal cortex in task inhibition also comes from electrophysiological studies. An increased negativity at parietal sites has been reported when switching back to a recently executed task than when switching to a less recent task [27–28]. More generally, the hypothesis that the parietal cortex is involved in overcoming task inhibition during task switching fits well with findings showing the involvement of parietal cortex in conflict resolution (e.g., [20]). For instance, the neural activity in posterior parietal cortex has been shown to vary with a physiologic index of conflict in competing processing neural pathways and to predict an enhanced behavioral adjustment [20].

However, cognitive and neural processes that mediate the overcoming of inhibition remain largely unclear. Evidence exists that task inhibition can be observed when interference between competing task sets occurs at the stimulus processing level (e.g., [42–44]) as well as when it occurs at the response level (e.g., [45,46]). Several studies suggest that posterior parietal cortex is anatomically well suited to detect stimulus conflict (e.g., stimuli eliciting multiple tasks), as it receives input from the extrastriate visual cortex and sends projections to lateral prefrontal cortex [47]. Previous studies have also emphasized a role for posterior parietal cortex in facilitating goal-directed attention to task-relevant aspects of a visual stimulus [48,49]. Thus, overcoming task inhibition could be mediated by biasing attention processes toward the current stimulus set, enhancing processing of task-related stimulus dimensions. Alternatively, the parietal cortex may support overcoming of inhibition by enhancing control over response-related features of the task set, for instance by enhancing representations of category-response rules (e.g., [50]). Evidence for involvement of parietal cortex in representations of stimulus-response associations or action rules do exist [50,51]. However, parietal cortex has been also involved in stimulus categorization (e.g., [20]). Since in our present procedure the task conflict occurred at both stimulus and response level (i.e., stimuli could elicit all the three possible tasks and the same motor responses were used for all the three possible tasks) it is not possible here to disentangle whether the role of parietal cortex in overcoming persistent inhibition is related to stimulus processing or to response-selection. Future studies could investigate whether frontal and parietal tDCS differently affect stimulus-related and response-related aspects of task inhibition.

One limiting factor of the current study was that on-line changes of neural activity in prefrontal and parietal cortex were not directly assessed during tDCS. This leaves open the possibility that tDCS also affected neural activity of other cortical regions.

In summary, our results show that experimentally-induced alterations of neural activity via right anodal/left cathodal tDCS of the dorsolateral prefrontal cortex and parietal cortex modulates performance during task switching, supporting previous observations about the involvement of both parietal and frontal cortex in cognitive control of task set. Importantly, right anodal/left cathodal tDCS of the parietal cortex improves performance only when switching back to a recently inhibited task and that thus requires previous inhibition to be overcome. Right anodal/left cathodal tDCS of the prefrontal cortex improves performance during task switching in general, either when re-engaging in a inhibited task or when engaging in a non-inhibited task, thus without affecting the ability to overcome task inhibition. This suggests a different contribution of prefrontal and parietal regions in task switching, with parietal cortex being selectively involved in overcoming persistent inhibition and prefrontal cortex being more generally involved in the control of task set during task switching.
